# The electroacupuncture-induced analgesic effect mediated by 5-HT_1_, 5-HT_3_ receptor and muscarinic cholinergic receptors in rat model of collagenase-induced osteoarthritis

**DOI:** 10.1186/s12906-016-1204-z

**Published:** 2016-07-13

**Authors:** Byung-Kwan Seo, Won-Suk Sung, Yeon-Cheol Park, Yong-Hyeon Baek

**Affiliations:** Department of Clinical Korean Medicine, Graduate School, Kyung Hee University, 26, Kyungheedae-ro, Dongdaemun-gu, Seoul 02447 Korea

**Keywords:** Collagenase-induced osteoarthritis, Electroacupuncture, Analgesic effect, Analgesic mechanism, Serotonergic receptor, Cholinergic receptor

## Abstract

**Background:**

Osteoarthritis (OA) is an degenerative disease characterized by chronic joint pain. Complementary and alternative treatment such as acupuncture have been utilized to alleviate pain. The objective of this study was to investigate the analgesic mechanisms of electroacupuncture (EA) in the collagenase-induced osteoarthritis (CIOA) rat model.

**Methods:**

Four weeks after inducing CIOA by injecting collagenase solution into the left knee of 5-week-old male Sprague-Dawley rats, 2 Hz and 100 Hz EA on Zusanli (ST 36) was performed. The analgesic effect of EA was evaluated by the tail flick latency (TFL) and paw pressure threshold (PPT) tests. To investigate the analgesic mechanism, serotonergic and muscarinic cholinergic receptor agonists and antagonists were injected 20 min prior to EA and the resultant changes were evaluated by the TFL and PPT tests.

**Results:**

EA on Zusanli (ST 36) demonstrated an analgesic effect in the CIOA rat model. The 2 Hz EA treatment showed a significantly greater analgesic effect than the 100 Hz treatment. The analgesic effect of 2 Hz EA was not strengthened by 5-HT1, 5-HT2, 5-HT3, and muscarinic cholinergic receptor agonist pretreatment, was blocked by 5-HT1, 5-HT3, and muscarinic cholinergic receptor antagonist pretreatment, but not blocked by 5-HT2 receptor antagonist pretreatment.

**Conclusions:**

In the CIOA rat model, EA on Zusanli (ST 36) exhibited analgesic effects, and 2 Hz EA resulted in a significantly greater analgesic effect than 100 Hz EA. The analgesic effect of 2 Hz EA was reduced by pretreatment of 5-HT1 receptor, 5-HT3 receptor and muscarinic cholinergic receptor antagonists.

## Background

Osteoarthritis (OA) is one of the most prevalent chronic joint disease at present. It is characterized by loss of articular cartilage, osteophyte formation, subchondral bone change, and synovitis [1]. OA has varying effects on the individual and on society. OA patients, especially elderly patients, experience symptoms every day, resulting in a lower quality of life. From a societal perspective, the increasing financial costs of treatment and management of OA are a challenging problem. The healthcare costs associated with OA in the USA exceed $60 billion annually and can increase up to $185.5 billion [2], demonstrating the importance of more effective OA treatments. To date, as complementary and alternative options for various treatment modalities including medications, patient education, exercise and physical therapy, non-pharmacological alternative modailities such as acupuncture and electroacupuncture (EA) have been administered [3, 4].

Acupuncture has been widely used to alleviate many types of pain, particularly chronic pain [5]. Human and animal study models have shown that acupuncture-induced analgesia is mediated through various neurotransmitters, modulators, and related factors including β-endorphin, enkephalin, endomorphin, and dynorphin [6].

The analgesic effects of EA have also been established through many studies. Researchers have conducted clinical trials and animal model studies on neuropathic pain and collagen-induced arthritis and demonstrated the descending modulation of nociceptive processing [7–11]. The analgesic effects and its adrenergic or opioidergic mechanisms of EA in CIOA in vivo study was reported [12, 13], but the serotonergic and cholinergic roles in EA analgesia have not been fully clarified. Current study was designed to investigate which receptors agonists and antagonists were involved in the analgesic effect of EA in CIOA in vivo model.

## Methods

### Animals

Five-week-old male Sprague-Dawley rats weighing 200 mg were obtained from Samtaco (Osan, Korea) and housed under controlled temperature (22 ± 1°C), humidity (55 ± 5 %), and 1:1 light-dark cycle (light from 6 AM to 6 PM). All animals had free access to food and water. All experiments were approved and conducted under the guidelines of the International Association for the Study of Pain and the Institutional Animal Care and Use Committee of Kyung Hee University [14].

### Induction of collagenase-induced osteoarthritis

After one week of adaptation to the laboratory conditions, intra-articular collagenase injection was performed; 0.05 ml of 4 mg/ml collagenase solution (Clostridium histolyticum, type II; enzyme activity 425 U/mg) was injected into the left knee of all rats. Four days after the first injection, a booster injection was administered. The gross articular manifestations were assessed and histopathological and serological analyses were performed according to previous CIOA studies [12, 13]. Briefly, the severity of stiffness was scored on a scale of 0–4 in the affected articulation as a reflection of edema and movement impairment. Two independent examiners assessed gross articular manifestations in a blind manner. At the end of the fourth week, the rats were sacrificed for histopathological analysis. Six parameters, i.e., including loss of the superficial layer, erosion of cartilage, fibrillation and/or fissures, disorganization of chondrocytes, loss of chondrocytes, and cluster formation, were evaluated for the histological analysis. Serum from each subject was prepared for the measurement of COX-1, COX-2, PGE2 activity (data not shown).

### Behavioral test

After four weeks of induction of CIOA and adaptation in the laboratory room conditions, tail flick latency (TFL) and paw pressure threshold (PPT) tests were performed at baseline, 10, 20, 30, 45, 60, and 90 min after initiation of EA.

To evaluate the analgesic effect on the thermal stimuli, the TFL test was performed using the tail flick unit (Ugo Basile Model 7360, Comrio, Italy) [15]. The rat was fixed in a 5.3 cm diameter × 15 cm length holder and the proximal third portion of six parts of the tail was laid on the 50-W infrared light bulb. The time lapse between the onset of irradiation and the flick of the tail was measured on the unit. The mean time was calculated after three continuous measurements and expressed in seconds. TFL test was performed at baseline, 10, 20, 30, 45, 60, and 90 min after initiation of EA. The time of irradiation was limited at 20 s and the portion of the tail exposed to the light bulb was shifted to prevent thermal injury. The change of TFL was calculated as a percentage of change of tail flick latency. The increase in the degree of TFL change represents the analgesic effect on the thermal stimuli.

$$ \mathrm{The}\kern0.5em \mathrm{degree}\kern0.5em \mathrm{of}\kern0.5em \mathrm{T}\mathrm{F}\mathrm{L}\kern0.5em \mathrm{change}\kern0.5em \left(\%\right)=\frac{\mathrm{post}.\mathrm{E}\mathrm{A}\kern0.5em \mathrm{T}\mathrm{F}\mathrm{L}{\textstyle \hbox{-}}\mathrm{baseline}\kern0.5em \mathrm{T}\mathrm{F}\mathrm{L}}{\mathrm{baseline}\kern0.5em \mathrm{T}\mathrm{F}\mathrm{L}}\times 100 $$

For evaluation of the analgesic effect on the mechanical stimuli, a PPT test was performed [16]. Rats were gently held in the cap and the algesiometer device (modified Randall-Selitto test; Ugo Basile, Comerio, Italy) was applied to the dorsal surface of the hind paw. The mechanical device increased the pressure by gram units until the rat withdrew its paw. The mean pressure was calculated after three consecutive measurements and expressed in grams. With 10-s intervals, the PPT test was performed at baseline, 10, 20, 30, 45, 60, and 90 min after initiation of EA. The upper limit of pressure was 250g to prevent tissue damage. The increase in the mean pressure represents the analgesic effect on the mechanical stimuli.

### Electroacupuncture treatment

After four weeks of induction of CIOA and adaptation to the laboratory condition, EA was performed into Zusanli (ST36), located laterally from tibial tuberosity and caudally below knee joint on the anterior tibialis muscle. The Zusanli acupoint is generally used to alleviate pain in clinical trials and animal studies [10–13, 17]. Two disposable sterile stainless needles (0.25 mm diameter × 40 mm length) were inserted into Zusanli (ST 36) and another point 5 mm away from the selected point. The acupuncture needle was inserted to the depth of 5 mm and stimulated with a train pulse (0.3 ms, 0.07 mA) for 30 min by an electrical stimulator (Nihon Kohden). At first, EA was performed at 2 Hz and 100 Hz to compare analgesic effects at different frequencies. The frequency that showed a better analgesic effect was selected when conducting experiments on the analgesic mechanism of EA.

### Pretreatment with agonists and antagonists

To investigate the analgesic mechanism, the 5-HT1 receptor agonist 8-OH-DPAT (8 ODT) and antagonist spiroxatrine (SPROX), the 5-HT2 receptor agonist DOI (DOI) and antagonist ketanserin (KTSRN), the 5-HT3 receptor agonist m-chlorophenyl-biguanide (mCLBG) and antagonist ondansetron (ODSTN), and the muscarinic cholinergic receptor agonist neostigmine (NSTM) and antagonist atropine (ATRP) were dissolved in sterile 10 % DMSO (dimethyl sulfoxide) and intraperitoneally injected 20 min before EA.

### Statistical analysis

All results were expressed in mean ± standard error of mean. In nonparametric procedures, statistically significant differences (*p* < 0.05) were determined by Friedman’s rank test followed by Dunnett’s post-hoc test within a group, Mann–Whitney U test between two groups, and Kruskal–Wallis ANOVA followed by Dunnett’s post-hoc test among groups.

## Results

### The analgesic effect of EA and comparison according to latency (2, 100 Hz)

**Fig. 1:**
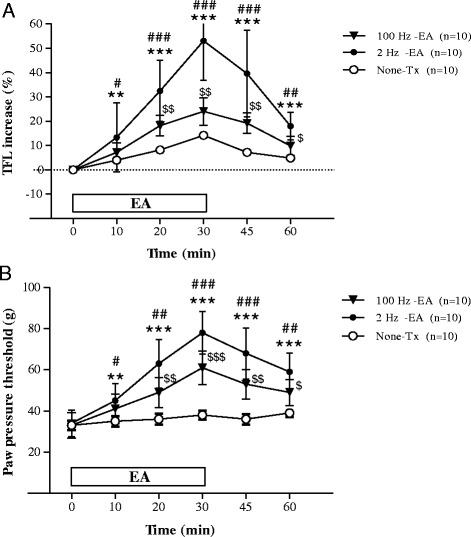
The effects of EA at 2 Hz and 100 Hz in the CIOA rat model assessed by TFL (**a**) and PPT (**b**). 2 Hz EA treatment group (2 Hz-EA, *n* = 10), 100 Hz EA treatment group (100 Hz-EA, *n* = 10) and no treatment group (None-Tx, *n* = 10). $*p* < 0.05, $$*p* < 0.01, $$$ *p* < 0.001: compared with None-Tx; **p < 0.01, ***p < 0.001: compared with None-Tx; #*p* < 0.05, ##*p* < 0.01, ###*p* < 0.001: compared with 100 Hz-EA

The effects of EA at 2 Hz and 100 Hz in the CIOA rat model are shown in Fig. 1. The degree of TFL change increased during 10–60 min and peaked at 30 min after initiation of EA. Both EA treatment groups showed statistically significant differences compared with the no treatment group (*n* = 10). The 2 Hz EA treatment group (*n* = 10) showed a significantly greater TFL change than the 100 Hz EA treatment group (*n* = 10) (Fig. 1a). PPT also increased during 10–60 min and peaked at 30 min after initiation of EA. Both EA treatment groups showed significant differences compared with the no treatment group (*n* = 10). Between the two EA treatment groups, the 2 Hz EA treatment group (*n* = 10) showed a significantly higher PPT than the 100 Hz EA treatment group (*n* = 10) (Fig. 1b).

### The 5-HT1 receptor Involvement of EA-induced analgesia

**Fig. 2:**
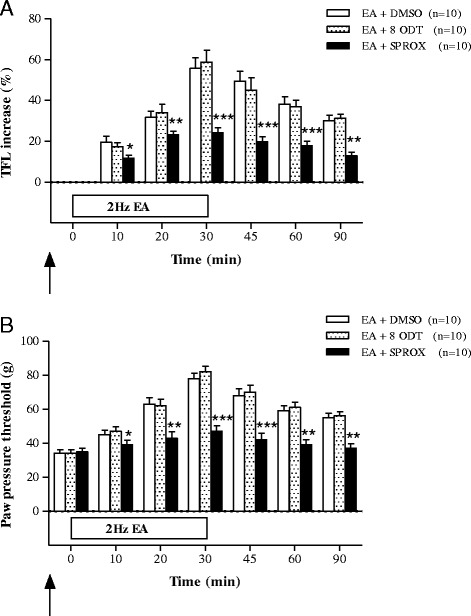
The effects of pretreatment of 5-HT1 receptor agonist (8-OH-DPAT, EA+8 ODT, *n* = 10) and antagonist (spiroxatrine, EA+SPROX, *n* = 10) in the CIOA rat treated by 2 Hz EA (EA+DMSO, *n* = 10) assessed by TFL (**a**) and PPT (**b**). Pretreatment with DMSO, 8 ODT, and SPROX was performed 20 min before 2 Hz EA. *p < 0.05, **p < 0.01, ***p < 0.001: compared with EA+DMSO

The effects of the 5-HT1 receptor agonist 8-ODT and antagonist SPROX on the analgesia induced by 2 Hz EA in the CIOA rat model are shown in Fig. 2. In the TFL test, there were no significant differences between the EA + 8 ODT group (*n* = 10) and the EA + DMSO group (*n* = 10). However, TFL increases induced by ST36 EA were significantly suppressed by SPROX pretreatment (*n*=10) 10–90 min after initiation of EA (Fig. 2a). In the PPT test, there were no significant differences between the EA + 8 ODT group (*n* = 10) and the EA + DMSO group (*n* = 10). However, PPT increases induced by ST36 EA were significantly suppressed by SPROX pretreatment (*n*=10) 10–90 min after initiation of EA (Fig. 2b).

### The 5-HT2 receptor Involvement of EA-induced analgesia

**Fig. 3:**
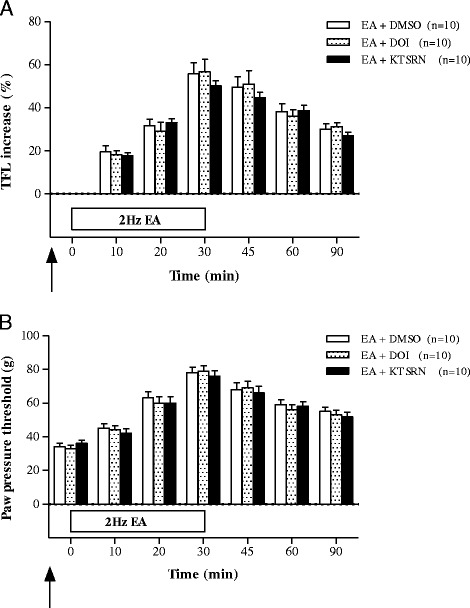
The effects of pretreatment of 5-HT2 receptor agonist (DOI, EA+DOI, *n* = 10) and antagonist (ketanserin, EA+KTSRN, *n* = 10) in the CIOA rat treated by 2 Hz EA (EA+DMSO, *n* = 10) assessed by TFL (**a**) and PPT (**b**). Pretreatment with DMSO, DOI, and KTSRN was performed 20 min before 2 Hz EA

The effects of the 5-HT2 receptor agonist DOI and antagonist KTSRN on the analgesia induced by 2 Hz EA in the CIOA rat model are shown in Fig. 3. In the TFL test, there were no significant differences between the EA + DOI group (*n* = 10), the EA + KTSRN group, and the EA + DMSO group (*n* = 10) (Fig. 3a). In the PPT test, there were also no significant differences between the EA + DOI group (*n* = 10), the EA + KTSRN group, and the EA + DMSO group (*n* = 10) (Fig. 3b).

### The 5-HT3 receptor Involvement of EA-induced analgesia

**Fig. 4:**
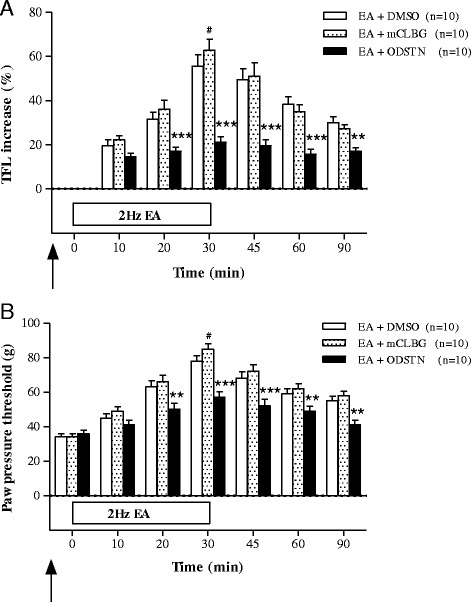
The effects of preatreatment of 5-HT3 receptor agonist (m-chlorophenyl-biguanide, EA+mCLBG, *n* = 10) and antagonist (ondansetron, EA+ODSTN, *n* = 10) in the CIOA rat treated by 2 Hz EA (EA+DMSO, *n* = 10) assessed by TFL (**a**) and PPT (**b**). Pretreatment with DMSO, mCLBG, and ODSTN was performed 20 min before 2 Hz EA. #*p* < 0.05, **p < 0.01, ***p < 0.001: compared with EA+DMSO

The effects of the 5-HT3 receptor agonist mCLBG and antagonist ODSTN on the analgesia induced by 2 Hz EA in the CIOA rat model are shown in Fig. 4. In the TFL test, there were no significant differences between the EA + mCLBG group (*n* = 10) and the EA + DMSO group (*n* = 10) except 30 min after initiation of EA. However, TFL increases induced by ST36 EA were significantly suppressed by ODSTN pretreatment (*n*=10) 20–90 min after initiation of EA (Fig. 4a). In the PPT test, there were no significant differences between the EA + mCLBG group (*n* = 10) and the EA + DMSO group (*n* = 10) except 30 min after initiation of EA. However, PPT increases induced by ST36 EA were significantly suppressed by ODSTN pretreatment (*n*=10) 20–90 min after initiation of EA (Fig. 4b).

### The muscarinic cholinergic receptor Involvement of EA-induced analgesia

**Fig. 5:**
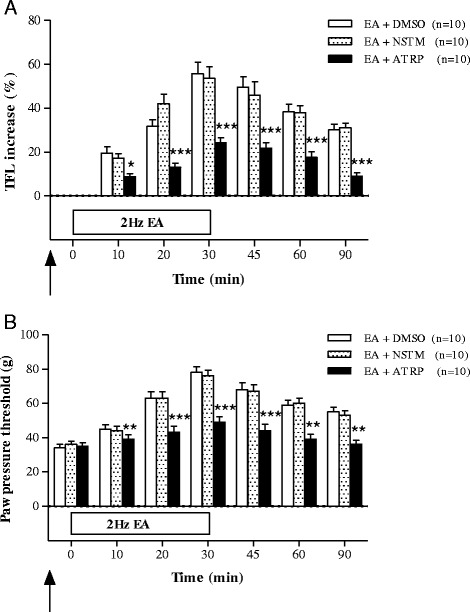
The effects of pretreatment of muscarinic cholinergic receptor agonist (neostigmine, EA+NSTM, *n* = 10) and antagonist (atropine, EA+ATRP, *n* = 10) in the CIOA rat treated by 2 Hz EA (EA+DMSO, *n* = 10) assessed by TFL (**a**) and PPT (**b**). DMSO, NSTM, and ATRP were pretreated 20 min before 2 Hz EA. *p < 0.05, **p < 0.01, ***p < 0.001: compared with EA+DMSO

The effects of the muscarinic cholinergic receptor agonist NSTM and antagonist ATRP on the analgesia induced by 2 Hz EA in the CIOA rat model are shown in Fig. 5. In the TFL test, there were no significant differences between the EA + NSTM group (*n* = 10) and the EA + DMSO group (*n* = 10). However, TFL increases induced by ST36 EA were significantly suppressed by ATRP pretreatment (*n* = 10) 10–90 min after initiation of EA (Fig. 5a). In the PPT test, there were no significant differences between the EA + NSTM group (*n*=10) and the EA + DMSO group (*n*=10). However, PPT increases induced by ST36 EA were suppressed by ATRP pretreatment (*n*=10) 10–90 min after initiation of EA (Fig. 5b).

### The involvement of each receptor agonists and antagonists in EA-induced analgesia

**Fig. 6:**
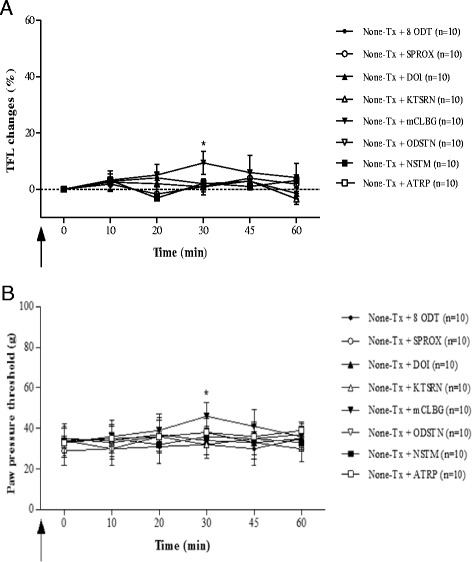
The effects of each receptor agonists and antagonists on the pain threshold in CIOA rat model assessed by TFL (**a**) and PPT (**b**). *p < 0.05: compared with each group

The effects of each receptor agonist and antagonist on the analgesia in the CIOA rat model are shown in Fig. 6. In both the TFL and PPT test, there were no significant differences between each receptor agonist and antagonist group except the None-Tx + mCLBG group at 30 min after measurement (Fig. 6).

## Discussion

OA is the most common degenerative joint disorder characterized by the progressive erosion of articular cartilage. The aching pain may worsen with use, and can be accompanied by morning stiffness, crepitus contributes to limited articular function and deteriorated quality of life [18]. The pathogenesis of OA is not entirely established, but is likely related to inflammatory cytokines that mediate cartilage destruction [19].

The current standard care for OA focuses on alleviating pain and managing symptoms. For the pharmacological treatment options, non-steroidal anti-inflammatory drugs (NSAIDs) have been considered as the primary therapy for OA [20]. Despite their universal administration for pain relief in osteoarthritis patients, the long term use of NSAIDs is controversial due to the gastrointestinal disorders and cardiovascular events related to their safety profile [21, 22]. The demands of osteoarthritis patients for non-pharmacologic therapies, especially acupuncture, have increased due to failure to alleviate pain and improve articular function.

EA has been used to treat a diverse range of painful conditions. Previous research has suggested a relationship between pain modulatory mechanisms and acupuncture analgesia, focusing on the role of transmitters and modulators [23]. The EA analgesia is initiated by needles triggering stimulation of afferent nerves and related with systemic activation of a variety of bioactive chemicals through peripheral, spinal and supraspinal mechanisms [24]. Research on EA analgesia induction and recovery profiles has demonstrated the possible involvement of humoral factors [25]. Several studies have been performed to prove the analgesic effect and mechanism of EA in various animal models, considering EA stimulation parameters such as frequency, intensity, and wave form [9–11]. However, there are limited studies on EA in CIOA model.

Various methods, including surgical procedures and intra-articular injections, can be used to induce OA. Intra-articular injection with chemical substances can be more conveniently performed in many studies because surgical procedures are complicated and take a longer period of time to induce degeneration [26]. When selecting a chemical substance, some studies have used papain but its use is limited by both its unclear mechanism of action and large dose requirement (4–12 mg) [27]. In contrast, a previous study demonstrated that CIOA was characterized by severe degenerative cartilage lesions, sclerosis of the subchondral bone below the cartilage erosions, osteophyte formation, and consequent deformity [28]. Other research showed that in the progression of OA, a larger amount of collagenase was detected [29] and cytokines stimulated the production of collagenase as a proteolytic enzyme [30]. In this regard, collagenase was considered to be appropriate to induce OA in rats that would be similar to the clinical manifestations in human version of OA.

In order to determine the proper time to conduct experiments with the CIOA model, the gross articular manifestations and histopathological and serological features were evaluated. In accordance with the results of previous studies, most of the osteoarthritic clinical and histopathological features were observed from 4 weeks after first collagenase injection. These features included altered pain-related behaviors, gross articular manifestations, cartilage-destructive features and serological biomarker activities [12, 13].

TFL and PPT tests are commonly used to evaluate the degree of nociception through the change of animal behavior. TFL is focused on the thermal stimuli and PPT is focused on the mechanical stimuli. Pain in OA is associated with thermal and mechanical hyperalgesia and affected by local mechanical and thermal factors [31]. TFL and PPT tests are appropriate to evaluate analgesic effects on thermal and mechanical stimuli in the CIOA, in accordance with other painful condition experiments [32]. However, there are few existing studies that have conducted TFL and PPT at the same time and showed positive results in the CIOA.

The acupoint Zusanli (ST 36) is traditionally used to reduce pain. Previous studies showed that EA on Zusanli (ST 36) led to analgesia using the tail flick method and c-Fos expression in the brain [33], and its effect was related to peripheral nerve receptors and biomarkers like β-endorphin and cortisol [34, 35].

Our results demonstrated that both 2 Hz and 100 Hz EA showed analgesic effect, evidenced in the TFL and PPT tests. The 2 Hz EA more effectively relieved thermal and mechanical hyperalgesia than the 100 Hz EA, in accordance with the results of previous studies. It has been shown that low-frequency EA is more effective for nociceptive pain whereas high-frequency EA is more effective for neurogenic pain [36]. Recent studies also showed that 2 Hz EA provides better and longer-lasting analgesic effects on mechanical allodynia [37, 38]. These results suggest that low-frequency EA is appropriate for the treatment of OA related pain.

In order to investigate the analgesic mechanism of EA, we conducted experiments with serotonergic and muscarinic cholinergic receptor agonists and antagonists. With respect to the serotonergic mechanism, our study showed that the analgesic effect of 2 Hz EA was not strengthened by the 5-HT1, 5-HT2, and 5-HT3 receptor agonists. In experiments with antagonists, the analgesic effect of 2 Hz EA was blocked by the 5-HT1 and 5-HT3 receptor antagonists, but not blocked by the 5-HT2 receptor antagonist. These results suggest that the 5-HT1 and 5-HT3 receptors partially mediate the analgesia induced by 2 Hz EA in the CIOA rat model. Ryu et al. [10] conducted experiments on the analgesic effects and mechanism of 2 Hz EA in the collagen-induced arthritis (CIA) model using 5-HT1A, 5-HT1B, and 5-HT4 receptor antagonists, and demonstrated that the analgesic effect of EA was blocked by 5-HT1A, 5-HT1B, and 5-HT4 receptor antagonists. Baek et al. [39] conducted experiments on the analgesic effects and mechanism of 2 Hz EA in a CIA model using 5-HT1A, 5-HT2, and 5-HT3 receptor agonists and antagonists, and demonstrated that the analgesic effect of EA was blocked by the 5-HT1A and 5-HT3 receptor antagonists, but not blocked by the 5-HT2 receptor antagonist. Chang et al. [40] demonstrated that 5-HT1A and 5-HT3 receptor antagonist blocked EA analgesia at three different frequencies (2, 10, and 100 Hz) and Kim et al. [41] suggested that 5-HT1A and 5-HT3 receptors had important roles in mediating the relieving effect 2 Hz EA on cold allodynia. In this regard, 2 Hz EA induces an analgesic effect through 5-HT1 and 5-HT3 receptors.

With respect to the cholinergic mechanism, our study showed that the analgesic effect of 2 Hz EA was not strengthened by the muscarinic cholinergic receptor agonist and blocked by muscarinic cholinergic receptor antagonist. These results suggest that the muscarinic cholinergic receptor partially mediates the analgesia induced by 2 Hz EA in the CIOA rat model. Baek et al. [39] also conducted experiments on the analgesic effects and mechanism of 2 Hz EA in the CIA model using a muscarinic cholinergic receptor agonist and antagonist, and demonstrated that the analgesic effect of EA was blocked by the muscarinic cholinergic receptor antagonist. Park et al. [42] studied the effects of 2 Hz EA on cold and warm allodynia in a neuropathic rat model using several muscarinic receptor antagonists and demonstrated that spinal muscarinic receptors, especially M1 subtype, mediate the EA antiallodynia. In this regard, 2 Hz EA induces analgesic effect through muscarinic cholinergic receptor.

Conflicting study results have been reported in the role of the serotonergic agonists and antagonists themselves in pain modulatioin. McCleane et al. [43] suggested that ondansetron could have an analgesic effect in neuropathic pain in a double-blind study and Ali Z et al. [44] showed that ondansetron reduced nociceptive response in behavioral and electrophysiological studies. Houde RW [45] showed the analgesic effectiveness of narcotic agonists and antagonists. Takagi et al. [46] examined the effects of several serotonin (5-HT) antagonists on 2 Hz EA analgesia in tooth pulp stimulation rat models and suggested that 5-HT1, except 5-HT1A; 5-HT2, except 5-HT2A; and 5-HT3 receptors are positively related to EA-induced analgesia.

The present study demonstrated that 5-HT antagonists ODSN intraperitoneal pretreatment suppressed the ST-36 2Hz EA-induced analgesia but there was no significant TFL and PPT changes when EA was not administered. It appears that 5-HT3 agonist mCLBG intraperitoneal pretreatment increased pain thresholds assessed by TFL and PPT not only in absence of EA treatment and also in ST-36 2Hz EA. Ali Z et al. [44] demonstrated that intrathecal administration of mCLBG increased the responsiveness of dorsal horn neurons to noxious stimulation. Sasaki et al. [47] reported that intrathecal 5-HT3 receptor agonist, 2-methyl-5-HT mediates antinociception to chemical stimuli.

In summary of the results of our research and previous studies, we speculate that the pain threshold-modulatory effects of serotonergic agonists and antagonits could depend on differences among the agonists, antagonists, time of agonists and antagonits pretreatment, administrative maneuver (intraperitoneal or intrathecal), and the type of pain models.

Considering limited knowledge on the therapeutical active components of EA and the physiological reactions followed by EA, restricted conclusive implication whether 2 Hz EA induced analgesia was mediated by serotonergic and cholinergic receptor mechanisms could be made. Because current study did not perform a comparison with the effect of non-acupoint EA, further studies are needed to investigate the acupoint specific effects of ST36 EA using various EA stimulation parameters including frequency.

## Conclusion

In summary, these observation suggest that 5-HT1, 5-HT3 and muscarinic cholinergic receptors partially mediate the analgesic effects of EA in CIOA. These results suggests that EA could be a potential option for the relieving osteoarthritic pain.

## Abbreviations

8 ODT, 8-OH-DPAT; ATRP, atropine; CIA, collagen-induced arthritis; CIOA, collagenase-induced osteoarthritis; DMSO, dimethyl sulfoxide; DOI, 5-HT2 receptor agonist DOI; EA, electroacupuncture; KTSRN, ketanserin; mCLBG, m-chlorophenyl-biguanide; NSAIDs, Non-steroidal anti-inflammatory drugs; NSTM, neostigmine; OA, osteoarthritis; ODSTN, ondansetron; PPT, paw pressure threshold; SPROX, spiroxatrine; TFL, tail flick latency
